# Binary Silanization and Silver Nanoparticle Encapsulation to Create Superhydrophobic Cotton Fabrics with Antimicrobial Capability

**DOI:** 10.1038/s41598-019-45622-0

**Published:** 2019-06-24

**Authors:** William Shen, Lishen Zhang, Xiaochun Li, Hua-Zhong Yu

**Affiliations:** 1 Department of Chemistry, Simon Fraser University, Burnaby, British Columbia V5A 1S6 Canada; 2 College of Biomedical Engineering, Taiyuan University of Technology, Taiyuan, Shanxi 030024 P.R. China

**Keywords:** Structural properties, Surface assembly

## Abstract

Cotton fabrics are functionalized with a binary solution of fluorine-free organosilanes and “encapsulated” with silver nanoparticles to achieve both superhydrophobic and antimicrobial properties. Derived from cellulose, cotton is one of the most abundant biologically generated materials and has been used in a wide variety of consumer goods. Nonetheless, cotton fabrics are not waterproof and prone to microbial contamination. Herein we report the rapid functionalization of cotton fabrics with a binary hexane solution of methyltrichlorosilane (MTS) and octadecyltrichlorosilane (OTS) at low concentration (0.17% v/v) followed by coating with colloidal silver nanoparticles (AgNP). The combined effects of binary silanization and AgNP encapsulation produced a surface that has remarkable water contact angle of 153 ± 2° and antimicrobial properties (against gram-negative *Escherichia coli)*. The superior performance of the modified cotton fabrics produced with fluorine-free organosilanes and silver nanoparticles augments the potential of improving the functionality of abundant biopolymers to be waterproof and contamination-resistant.

## Introduction

Superhydrophobic surfaces have long been known on a variety of surfaces in nature including both plants and animals^1^. For example, Barthlott *et al*. reported that some plants such as *Nelumbo nucifero* form epicuticular wax crystalloids that dramatically increase self-cleaning and water-repellent capabilities^2^. Likewise, the waterproof properties of water strider legs follow the same concept where cuticle wax is secreted with nanoscale roughness which allows them to stand on water^3^. Inspired by many of these natural phenomena, a wide variety of treatment techniques have been developed to turn hydrophilic surfaces such as glass, paper, polycarbonate, silicon dioxide, titanium, and zinc oxide into ones that are hydrophobic and even superhydrophobic, which has been reviewed by other groups^1,4^.

Another natural material, cotton has been popular to develop treatments for as it is one of the most abundant biopolymers found on Earth. Conversely, it is inexpensive and used in a wide array of consumer products. Unfortunately, cotton, due to its porous nature is easily wetted and contaminated. Moreover, cotton’s large pore volume causes it to swell from water retention^5^. The water retention capacity is further increased when cotton is scoured to remove naturally occurring wax and other non-cellulosic materials^6^. The reality is that water retention is not always desirable, therefore, the ability to convert cotton to possess a hydrophobic surface enables profound properties to be imparted namely water repellency and self-cleaning abilities.

In the past several years, a variety of techniques to generate these hydrophobic/superhydrophobic surfaces have been developed including hydrothermal, solution-immersion, chemical vapour deposition, electrospinning, layer-by-layer assembly, and pad-dry^7^. Of all these techniques, the solution-immersion method has many advantages such as efficiency, scalability, minimal equipment requirements, and cost-effectiveness. In addition to superhydrophobic properties, materials that are also antibacterial may find relevant applications in packaging, biomedical, and other textiles industries. Silver, a popular antibacterial agent used in textile-related applications has been demonstrated to show broad activity towards various bacteria, yet low activity towards mammalian cells^8^. The phenomenon has been a main reason why silver has been highly popular for use in items such as prosthetics, catheters, and wound dressings^9^.

Herein, we describe a rapid, one-step immersion process with a binary, fluorine-free silane solution for modifying cotton textiles to attain superhydrophobic, and to achieve antimicrobial capability by subsequent encapsulation with silver nanoparticles. In particular, by implementing a binary methyltrichlorosilane (MTS) and octadecyltrichlorosilane (OTS) mixture, we are able to significantly decrease the amount of silanes and the reaction time required for achieving superhydrophobicity compared with traditional silanization processes.

## Results and Discussion

### Binary silanization to create superhydrophobic cotton fabrics

Modification of cotton fabrics with a binary mixture of MTS and OTS has been confirmed to increase the hydrophobicity noticeably in comparison to either MTS or OTS alone. Variations in the ratio of MTS to OTS used in the coating solution, reaction time, and total silane concentration are all crucial to obtaining a superhydrophobic surface. For all samples treated with a mixture of MTS and OTS, the relative humidity was maintained at approximately 50% during sample preparation; it has been confirmed that relative humidities in this range are beneficial for silane diffusion and in promoting faster hydrolysis and polycondensation of the precursors^10^.

**Figure 1:**
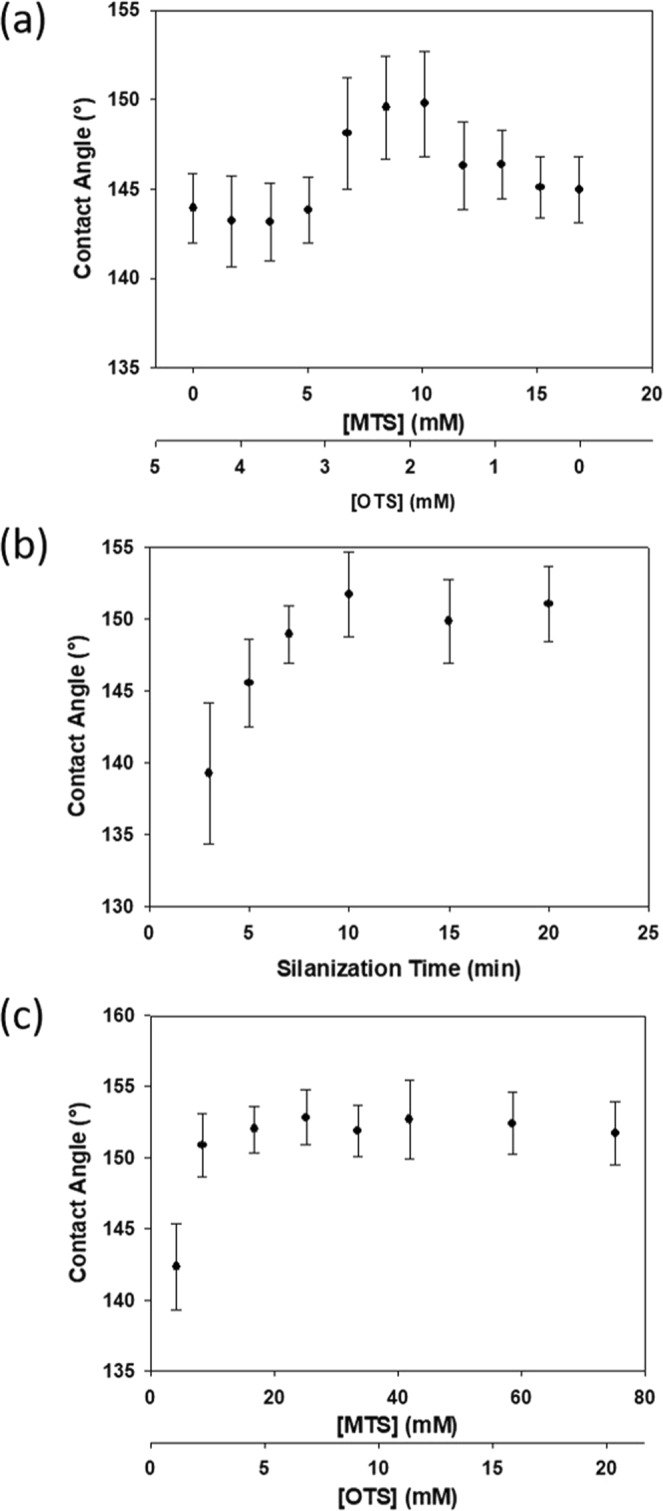
Contact angle measurements of cotton fabrics treated with (**a**) different ratios of MTS and OTS, (**b**) different silanization times using 8.4 mM MTS and 2.3 mM OTS, and (**c**) increasing the concentrations of both silanes while maintaining a ratio of 4:1 MTS:OTS.

Based on our previous experimental observation on other substrates (*e.g*., glass slides^11^, laboratory filter paper^12^), initial treatments on cotton to establish the modification protocols used 15–40 mM MTS or OTS for a reaction period of 5–10 min to produce water contact angles (WCA) of 145 ± 2°. While both MTS and OTS treatments had similar high but below superhydrophobic contact angles (<150°), we were motivated to try MTS and OTS together in a mixture (*vide supra*). The enhanced hydrophobicity of using a binary silane mixture has been previously reported on paper substrates only^12^. Having tried different ratios, it was observed that when a 4:1 ratio of MTS and OTS (with a total concentration of 10 mM; 0.14% v/v of MTS and 0.09% v/v of OTS) were used, the contact angle increased to 150 ± 3° (Fig. 1a). The main benefit of using a mixture of MTS and OTS was to achieve the superhydrophobicity, for which neither OTS nor MTS treatment would be able to. Another benefit is that much less silanes were needed than the single-component silanization processes. Importantly, we have discovered that treatments involving greater than 20 mM OTS can damage the cotton fabrics making them fragile with solid particles visible to the naked eye (Supplementary Information).

To optimize the process, we examined the impact that different durations of the silanization reaction would have on the resulted hydrophobicity. When the silanization time was increased to 10 min, the contact angle increased slightly to 152 ± 3° (Fig. 1b). Khoo *et al*. observed that increased reaction time of MTS on glass produced thicker polymethylsiloxane films of nanofibers^13^. The marginal increase in contact angle, thus, was likely due to an increase in the coverage of the film formed from silanization given that more time was allotted for the hydrolysis and condensation reactions to proceed. Longer reaction durations than 10 min did not show an appreciable increase in water contact angle. Particularly, when we silanized cotton with OTS for 3 h there was no significant difference in hydrophobicity compared to 10 min. The lack of notable improvements suggests that the coating film had reached sufficient roughness and coverage to prevent water from directly contacting the bare cotton fabrics. Not surprisingly, the opposite effect (*i.e*., decrease in the contact angle) occurred when the silanization duration was shortened.

Subsequently, we examined the effect of increasing the total silane concentration while maintaining the optimized ratio (4:1 for MTS:OTS) with a fixed reaction time (10 min). A three-fold increase in the concentrations of MTS and OTS showed a marginal increase in the contact angle (153 ± 2°) and no further increases were observed thereafter. Halving the concentration of each silane component equally (a total concentration of 5 mM) caused a decreased water contact angle (142 ± 3°), indicating that there were not enough silane species to form a full coating on the cotton fabrics. The results of the optimized mixture of silanes were striking as cotton treated with only OTS using similar optimization schemes also produced a large water contact angle, albeit at the expense of a much higher concentration (>50 mM). Moreover, with high concentrations of OTS to treat the surface, we can observe the accumulation of large silicate particles that are not mechanically stable on the surface (*i.e*., can be easily removed by abrasion).

**Figure 2:**
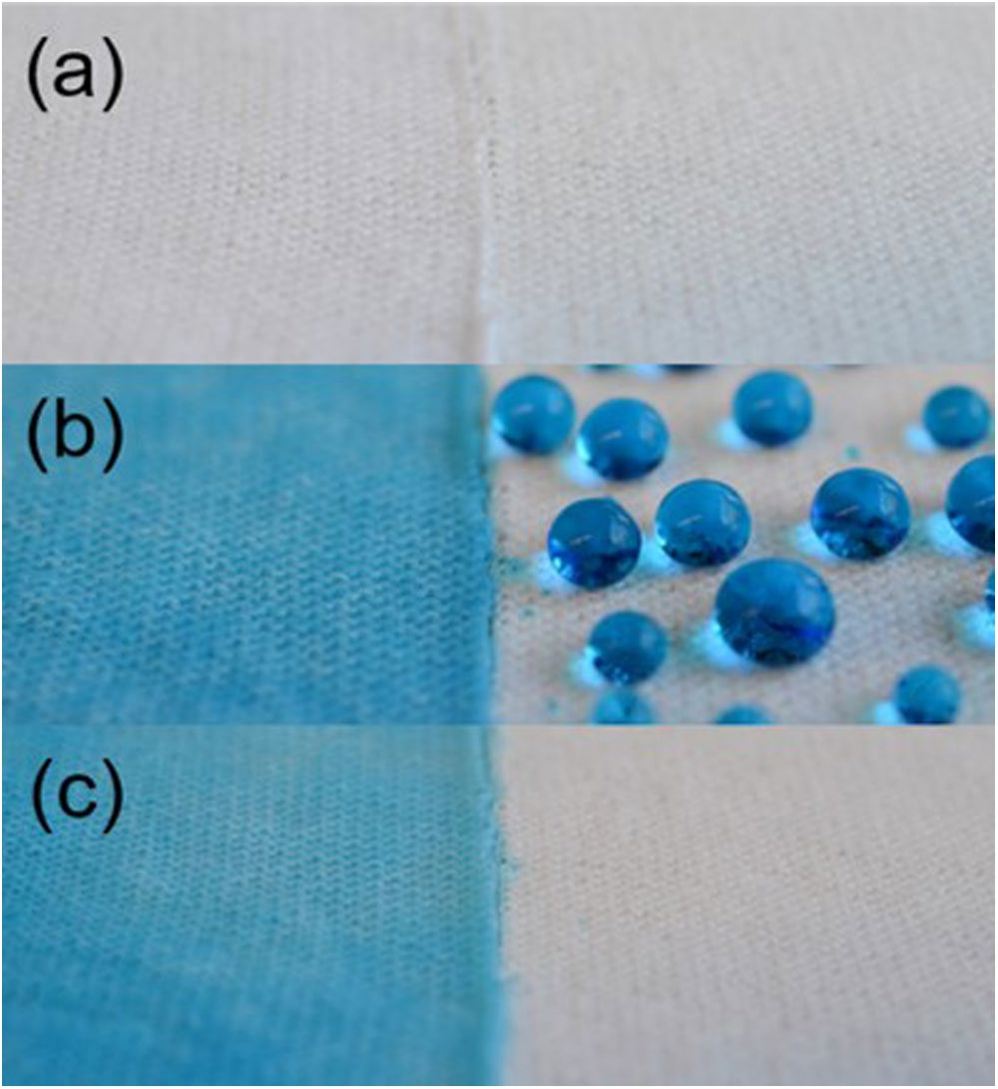
Cotton fabrics sprayed with blue dye solution where the samples on the left correspond to unmodified and the right corresponds to treated with MTS/OTS. (**a**) bare cotton, (**b**) suface sprayed with blue dye (**c**) the surface was blown briefly with nitrogen gas.

To visualize the impact of binary salinization of the cotton fabrics, we took both unmodified (Fig. 2a, left section of the sample) and MTS/OTS modified cotton (Fig. 2a, right section of the sample) and sprayed the surface with a blue dye. On the unmodified surface, the dye wicked into the cotton almost immediately while the liquid on the MTS/OTS sample remained on the surface (Fig. 2b). After blowing both surfaces with a quick stream of nitrogen gas, the droplets of dye on the treated cotton were removed immediately while the absorbed liquid in the untreated cotton remained unaffected (Fig. 2c). The lack of any dye remaining on the MTS/OTS surface reaffirms the water repellent properties that the treatment creates.

The presence of variations in the measured contact angles across the sample surface (*i.e*., rather large standard deviation) suggests that the coating on the surface is non-uniform. Uniformity would be difficult to achieve on such a macroscopically rough and porous surface compared to relatively flat substrates such as silicon dioxide^10,14^ and titanium^15^. Furthermore, modification schemes for these types of surfaces have generally entailed only a single type of silane such as MTS or OTS rather than a mixture. Due to the length dependency of the alkyl chain on polymerization (condensation of the silanols)^10,16^, OTS with its rather long octadecyl chain would be expected to react slower than MTS^14,15^. As our treatment consists of a mixture of MTS and OTS, MTS would inhibit OTS from undergoing horizontal “polymerization” due to its higher reactivity and ability to form three-dimensional aggregates^13,17^.

### Encapsulation with Ag nanoparticles to achieve antimicrobial capability

After obtaining the ideal conditions required for superhydrophobic performance for the cotton fabrics, we impregnated the surfaces with a suspension of 30 ppm AgNPs to examine if the surface was able to retain water repellency while incorporating antimicrobial properties. We synthesized the AgNP using the Creighton method which involves the reduction of AgNO_3_ with NaBH_4_^18–20^. The nanoparticles produced had a surface plasmon resonance (SPR) peak at 391 nm with a narrow particle size distribution centered around a diameter of 15 nm as characterized by UV-Vis spectroscopy and dynamic light scattering (DLS) measurements, respectively (Supplementary Information). AgNP with small diameters are correlated with increased antimicrobial activity as they have a higher surface area to volume ratio available to interact with microbes^21,22^ and can also diffuse quicker^23^.

**Figure 3:**
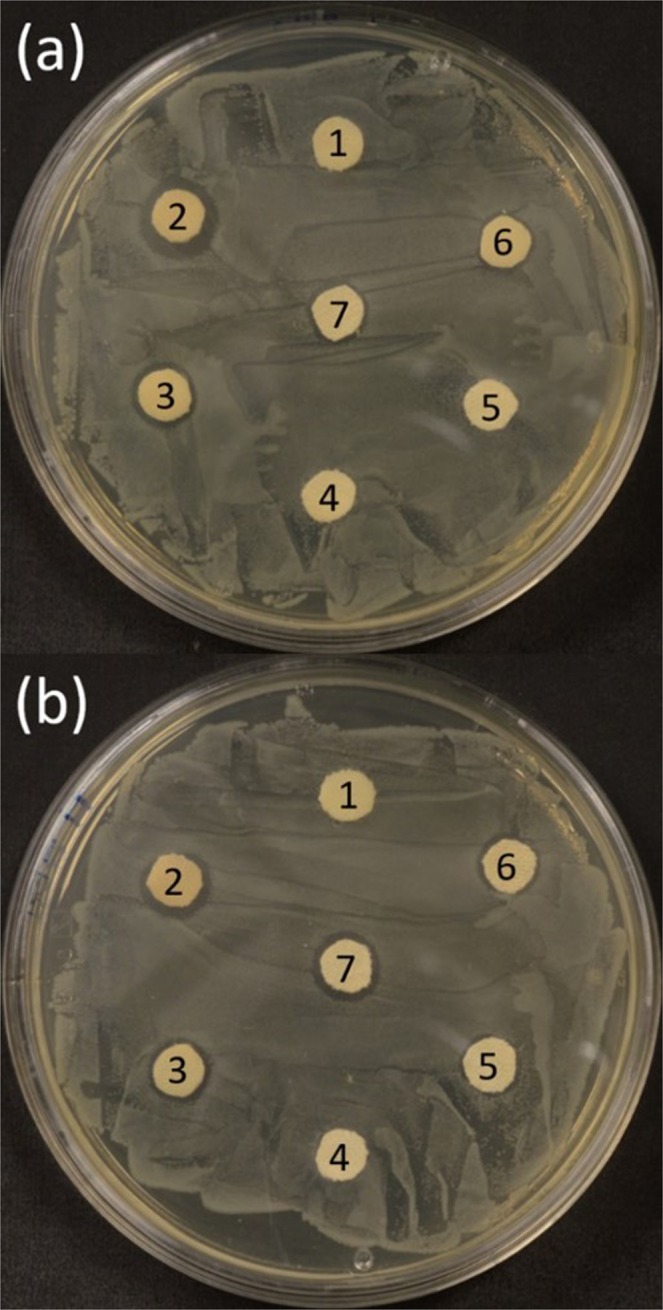
Kirby-Bauer disk susceptibility test using *E. coli* where (**a**) compared treatment methods and (**b**) compared silanization times for MTS/OTS/AgNP. Disks 1–4 for all plates were the same with untreated (1), AgNO_3_ (2), AgNP (3), and MTS/OTS (4). The remaining disks for (**a**) were as follows: MTS/OTS then immersion in AgNP (5), immersion in AgNP then MTS/OTS (6), and MTS/OTS/AgNP (7). Plate (**b**): 5 min MTS/OTS then AgNP spray (5), 7 min MTS/OTS then AgNP spray (6), and 10 min MTS/OTS then AgNP spray (7).

For the antimicrobial treatment of MTS/OTS cotton, we opted to treat the surfaces with AgNP using various methods. The trials included reacting silane with the surface followed by AgNP immersion, AgNP immersion followed by silanization, and silanization followed by spraying AgNP onto the surface (MTS/OTS/AgNP). To characterize the antimicrobial activity of the materials, a modified version of the Kirby-Bauer disk susceptibility assay was employed with gram-negative *E. coli* as the test organism^24^. Out of all three treatment methods, only the two methods involving silanization followed by treatment with AgNP demonstrated zones of inhibition (ZOI) which indicated the presence of antimicrobial activity (Fig. 3a). Both untreated and MTS/OTS cotton did not show the presence of ZOI so we attributed the growth inhibition in the AgNP samples to be solely due to the nanoparticle’s presence. Treatment with AgNP only was observed to have a similar sized ZOI as MTS/OTS/AgNP suggesting that the silane polymer does not impede on antimicrobial activity. Bare cotton treated with silver nitrate caused a ZOI that was larger than those caused by AgNP which agrees with the literature as the toxicity is believed to be caused primarily by silver ions which AgNP must first oxidize to^21,22,25,26^. The silver ions that are generated have the ability to interact with thiols found in proteins resulting in their inactivation^27^. In regards to the nanoparticles themselves, there is much debate as to exactly how much they contribute towards toxicity.

We did not observe a ZOI when treating unmodified cotton first with AgNP followed by silanization (Fig. 3a). The lack of a ZOI may be due to aggregation of the nanoparticles during the silanization procedure which would lead to a decrease in antimicrobial activity^28,29^. In addition, the formation of the polymeric coating on top of nanoparticles may trap them and prevent them from interacting with a bacterial cell membrane in an appreciable level^30^. We examined the effect of treating samples of cotton with our silane mixture for 5, 7, and 10 min followed by spraying with AgNP (Fig. 3b). As the silanization period was decreased from 10 min, the ZOI was also decreased to the point where at 5 min, was hardly observable. As discussed previously, a shorter silanization time could cause thinner and incomplete formation of the coating. When treating the silanized cotton with colloidal silver, the reaction rate of any unreacted MTS and OTS would increase as the AgNPs were dispersed in water. The more water is available, the quicker silanol cross-linking will occur that could potentially “encapsulate” AgNPs within the three-dimensional polymethylsiloxane networks. It was confirmed that the superhydrophobic performance of AgNP impregnated cotton by the spray method (MTS/OTS/AgNP) was not hindered as the measured contact angle was as high as 151 ± 1°. In other words, samples treated with MTS/OTS/AgNP had a marginal decrease in hydrophobic performance over strictly MTS/OTS treated samples.

### Structural and morphological characterization of functionalized cotton fabrics

**Figure 4:**
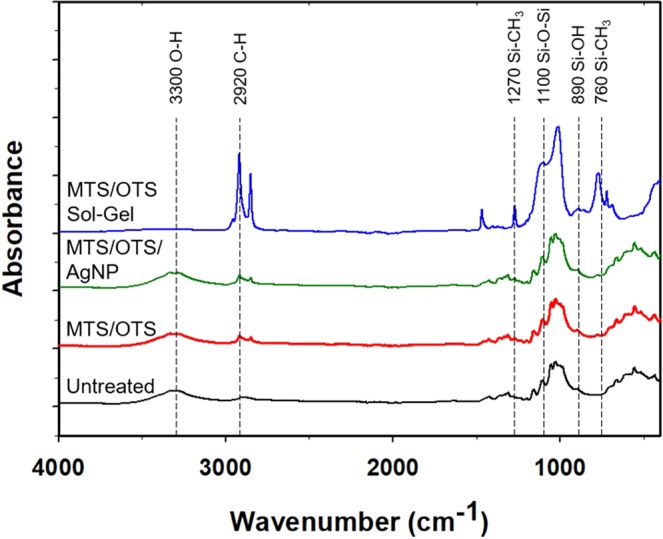
ATR FT-IR of untreated cotton (black trace), MTS/OTS cotton (red trace), MTS/OTS/AgNP cotton (green trace), and a pure sol-gel derived from a mixture of MTS and OTS (blue trace).

**Figure 5:**
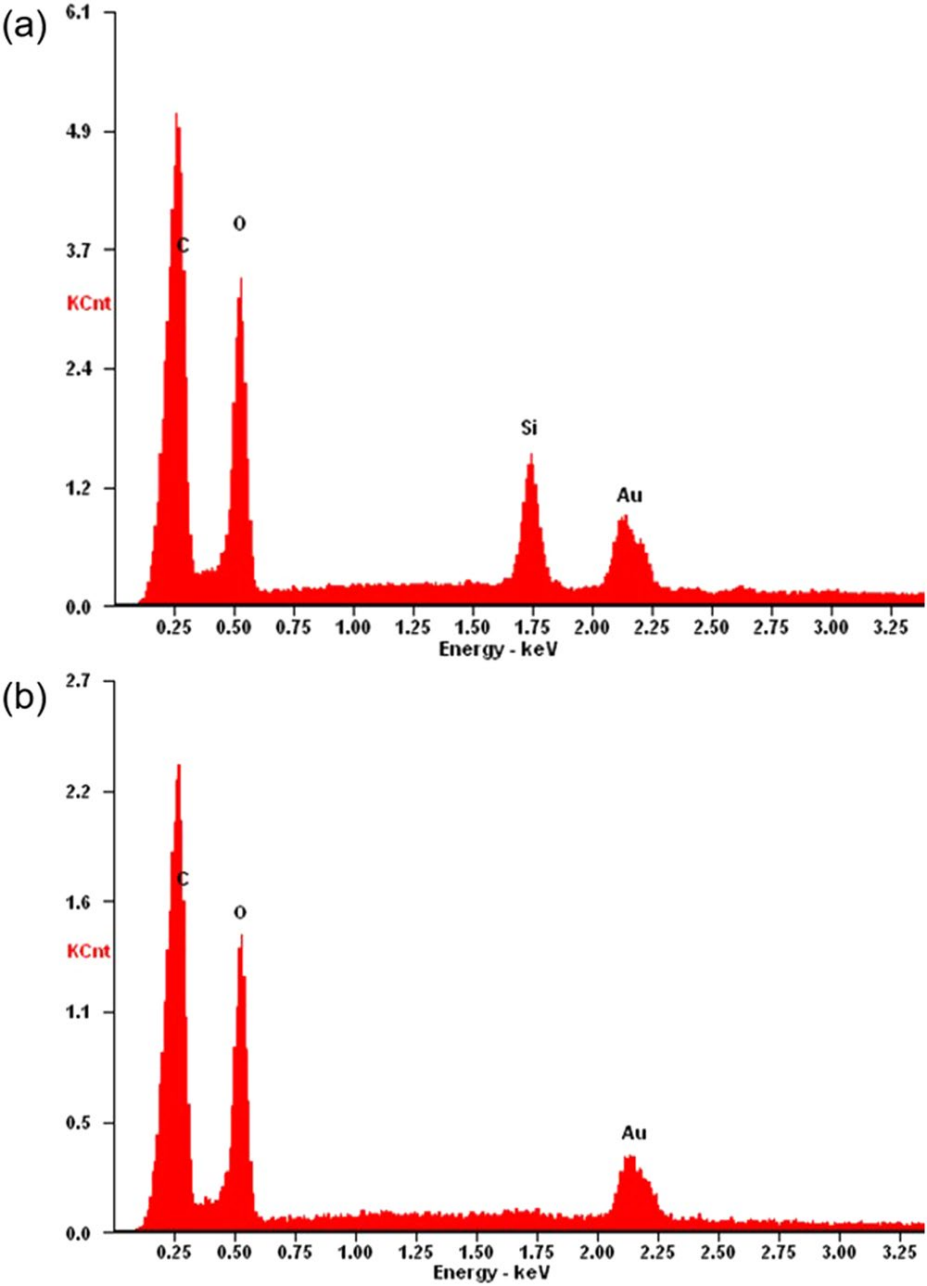
EDS spectra of (**a**) MTS/OTS/AgNP and (**b**) untreated cotton. The Si K_α_ peak is present at 1.74 keV indicating presence of Si species.

To gain a better understanding of the coating resulting from the binary silanization and AgNP encapsulation, we performed ATR FT-IR and energy-dispersive X-ray spectroscopy (EDS) to probe the surface before and after treatment. All FT-IR spectra displayed a C-H stretch at 2920 cm^−1^ which indicated the presence of methyl groups (Fig. 4). When comparing pure MTS/OTS sol-gel to the coated samples, the C-H stretch had the largest absorbance due to the abundance of methyl and octadecyl chains. On the other hand, the spectrum did not show the presence of an absorption band at 3300 cm^−1^ indicating that the sample did not contain a detectable amount of hydroxyl groups. As the untreated and treated samples had similar absorbances at 3300 cm^−1^, we examined the peak at 890 cm^−1^ (Si-OH). The untreated and treated samples had no difference in absorbances at this position suggesting that the silanized cotton samples do not contain an appreciable amount of free silanol groups. The treated samples also contained a small absorbance peak at 1270 cm^−1^ indicating the presence of Si-CH_3_ bonding. However, the corresponding absorbance for Si-CH_3_ was not detected at 760 cm^−1^ and may be attributed to the low abundance of the polymethylsiloxane coating in relation to the cellulose substrate. The treated samples showed a peak at 1100 cm^−1^ which may correspond to Si-O-Si bonds. Further analysis with EDS revealed the presence of a Si K_α_ peak at 1.74 keV (Fig. 5a) that was absent in the untreated samples (Fig. 5b).

**Figure 6:**
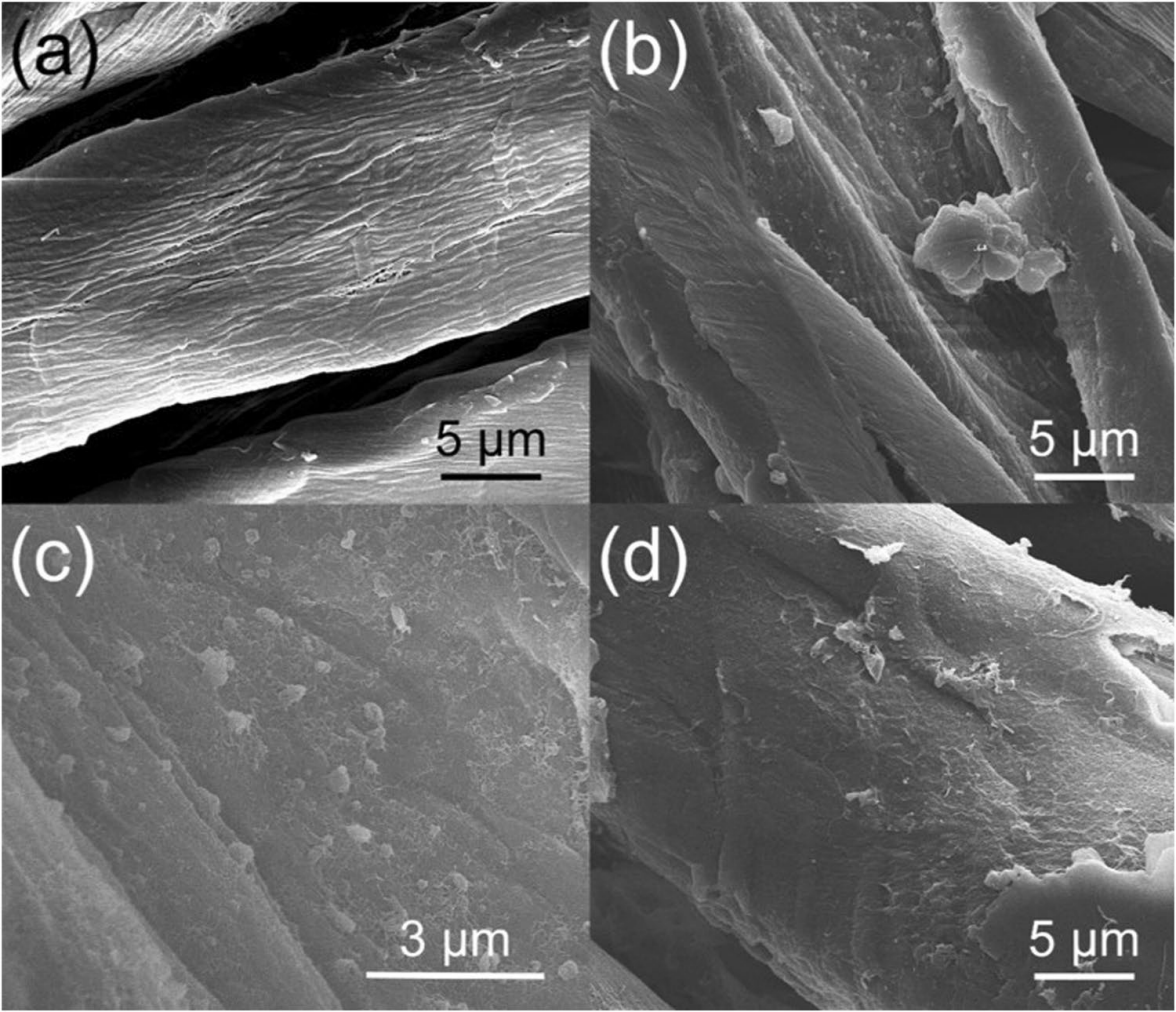
SEM images of (**a**) untreated, (**b**,**c**) MTS/OTS, and (**d**) MTS/OTS/AgNP treated cotton fabrics.

To examine the morphology of the modified cotton fabrics, scanning electron microscopy (SEM) studies were performed. Treatment with MTS/OTS caused an increase in the roughness of the surface due to the formation of both “nanospheres” and “nanofibers” (Fig. 6b,c), both features were not observed on untreated cotton (Fig. 6a). Similar nanosized features were previously reported by Khoo *et al*. to be formed upon treatment of glass with only MTS^13^. Thus, it is possible that the MTS in our silane mixture, with its higher reactivity, undergoes fast hydrolysis and condensation, which contributes primarily to the formation of these nanostructures. Treatment with MTS/OTS/AgNP similarly also formed nanofibers, however, nanospheres were not observed (Fig. 6d). Rather, some areas of cotton fibers were coated with “nano-islands” that are smooth in appearance. The islands likely formed during the addition of AgNP immediately after silanization as the water content would increase the rate at which silane reacts with each other rather than the surface of the substrate. Treatment of cotton with a coating solution of only OTS/AgNP created intricate structures with large increases in both micro and nanoscale roughness. The increase in roughness included contributions from both fibrillar and spherical particles, both of which can increase the water contact angle^31^. The OTS/AgNP treated samples were noticeably more rough than MTS/OTS/AgNP, however, the water contact angles of both treatment methods were similar with angles of 148 ± 2° and 151 ± 1°, respectively. Moreover, treatment with OTS/AgNP required a much higher concentration of silane compared to MTS/OTS/AgNP and large aggregates of silane were even visible with the naked eyes.

### Comparison with state-of-the-art functionalization protocols

In retrospect, a handful of protocols have been developed to functionalize cotton fabrics to possess both superhydrophobic and antimicrobial characteristics (Table 1)^32–44^. Different precursors such as organosilanes^32–36^, silica sols/particles^37,38^, organoacids^39,40^, polymers^41,42^, and metal particles^43,44^ have been utilized for creating the superhydrophobic surfaces. Among them, fluorine-free organosilanes shall be highlighted because they are inexpensive and can form stable “coatings” via chemical bonding on the surface^10–12^. For the formation of antimicrobial characteristic, metal particles containing silver^32–36,38,40,42–44^, copper^37,39^, and zinc^24^ were the choices. Silver nanoparticles are of particular interest for practical applications, because of their nontoxic characteristics and ease of preparation. However, most of these established protocols (including conventional silanization reactions^32–36^) require the treatment time up to hours and high concentration of the reagents; for the others, multistep precursor preparation and disposition were necessary.

**Table 1:** Comparison of the binary salinization/AgNP encapsulation process with the literature methods for preparing superhydrophobic cotton fabrics with antimicrobial capability.

Ref.	Author(s)*	Superhydrophobic modification	Antimicrobial formulation	WCA
This work	H.-Z. Yu	Immersion in a binary CH_3_(CH_2_)_17_SiCl_3_ (0.14% v/v)/CH_3_SiCl_3_ (0.09% v/v) for 10 min	Subsequent formation of AgNP by reducing AgNO_3_ with NaBH_4_	153 ± 2°
^32^	M. Shateri Khalil-Abad	Immersion in CH_3_(CH_2_)_7_Si(OC_2_H_5_)_3_ (3% v/v) in ethanol/water for 18 h	Prior formation of AgNP by reducing AgNO_3_ with ascorbic acid	151°
^33^	C.-H. Xue	Immersion in CH_3_ (CH_2_)_15_Si(OCH_3_)_3_ (3% v/v) in ethanol for 1 h	Prior formation of AgNP by reducing [Ag(NH_3_)_2_]^+^ with glucose	157.3 ± 1.6°
^34^	R. Guo	Immersion in H_2_N(CH_2_)_3_Si(OCH_3_)_3_; 1% (vol.) in acetone for 24 h	Subsequent formation of AgNP by reducing AgNO_3_ with sodium citrate	153°
^35^	M. Shateri-Khalilabad	Immersion CH_3_(CH_2_)_7_Si(OC_2_H_5_)_3_ 1% (vol.) in ethanol/water (9:1) for 24 h	Prior formation of AgNP by reducing AgNO3 with ascorbic acid on NaOH treated surface	156 ± 3.8°
^36^	M. A. Kulandainathan	Immersion in H_2_N(CH_2_)_3_Si(OC_2_H_5_)_3_; 0.25% (w) in EtOH:NH_4_OH:H_2_O (1:0.66:1) for 8 h followed by encapsulation with poly(styrene-co-maleic anhydride) (PSMA)	Subsequent formation of AgNP by reducing AgNO_3_ with triethylamine	>150°
^37^	R. Khajavi	Immersion in silica sols with subsequent treatment with CH_3_(CH_2_)_15_Si(OCH_3_)_3_	Doping the preformed silica sols with Cu NPs	151.1 ± 0.3°
^38^	C. F. Guo	Silica nanoparticle (NP)/polydimethylsiloxane (PDMS) coating on one side	A cellulose acetate/acetone/IL solution coated on the other side, a silver film is then thermally deposited	161 ± 2°
^39^	M. G. Sethuraman	Immersion of Cu-coated surface in 0.1% stearic acid for about 12 h	Immersion in a solution of copper acetate and ascorbic acid for 1 h	159°
^40^	H. Li	Post-modification with HS(CH_2_)_10_COOH/HS(CH_2_)_11_CH_3_ (0.16% w)	Coating with polydopamine, followed by *in situ* deposition of Ag NP	153°
^41^	X. Liu	Post inclusion of an epoxy alkane/epoxy cross-linker	Depositing polyhexamethylene biguanide	>150°
^42^	J. Sun	Post modification with fluorinated decyl polyhedral oligomeric silsesquioxane (F-POSS)	Sequential deposition of branched poly(ethylenimine) (PEI) and AgNPs	169°
^43^	M. G. Sethuraman	Bimetallic deposition of copper and silver over cotton fabric		160.9°
^44^	M. Shaban	Spin coating with zinc oxide (ZnO) NP prepared by sol-gel method		154 ± 1°

As has been characterized in detail and compared with previous established procedures (Table 1), our one-step immersion process with a binary, fluorine-free silane solution for modifying cotton textiles to attain superhydrophobicity (and achieving antimicrobial properties with subsequent encapsulation with silver nanoparticles) has the potential to fulfill the requirements for practical applications. In particular, the implementation of a binary MTS and OTS solution, we were able to significantly decrease the amount of silanes required (total silane concentration is 0.17% v/v) while attaining comparable superhydrophobicity as traditional silanization processes^32–36^. Compare to other non-silanization based methods^37–44^, this method required no pre-treatment of coating precursor and can accomplish in less than 10 min. Both fluorine-free sliane precursors have been in mass production for decades; the use of low concentration of silanes allows for not only an associated reduction in costs but also enables the modification procedure to be performed rapidly and potentially at large scales without impacting the environments and ecosystem. Contact angle measurements showed negligible differences in superhydrophobic performance between cotton surfaces upon binary silanization and after AgNP encapsulation confirms the success of the implementation of both superhydrophobicity and antimicrobial capability at the same time.

## Conclusions

Cotton fabrics can be functionalized to attain both superhydrophobic and antimicrobial properties via a unique binary silanization process in conjunction with silver nanoparticle encapsulation. The use of a binary mixture of long and short silanes (OTS and MTS) was demonstrated to achieve a high level of superhydrophobicity and requires significantly less reactants when compared with traditional silanization processes. Additionally, the solution immersion treatment was simplistic in nature with the reaction time of minutes; it is beneficial to the environment over traditional coatings as it utilizes non-fluorinated silanes. Future experimental investigation would open new avenues to include the treatment of a wide variety of different surfaces in addition to cotton fabrics such as synthetic fabrics including polyesters and polyamides. The use of other species such as zinc nanoparticles to impart antimicrobial properties will also be explored to replace noble metals. Beyond the scope of this work, this simple and facile reaction could be potentially scaled for industrial applications after addressing challenges such as choosing appropriate solvents, designing suitable vessels, and developing feasible waste disposition protocols.

## Experimental

### Materials and reagents

Methyltrichlorosilane (MTS, ≥99%) and octadecyltrichlorosilane (OTS, >90%) were purchased from Sigma-Aldrich (St. Louis, MO). Toluene is of ACS reagent grade and ordered from Fisher Chemical (Fair Lawn, NJ). AgNO_3_ (99%) was purchased from Anachemia (Montreal, QC). NaBH_4_ (12% w/w, in 14 M NaOH) was obtained from Aldrich as well. Bleached woven cotton fabric (16.5 mg/cm^2^) was purchased locally. All aqueous solutions were prepared with deionized water (>18.2 MΩ • cm), produced with a Barnstead EASYpure UV/UF compact water system (Dubuque, IA).

### Surface silanization and nanoparticle encapsulation

The cotton fabrics were first cut into 1 × 3 cm^2^ pieces, cleaned ultrasonically in deionized water for 30 min and dried before use. The silanization reaction for modifying cotton fabrics was performed by immersion in a coating solution prepared by adding a specified amount of MTS and/or OTS into 10 mL of toluene in a sealed container under ambient conditions (20–22 °C, ~50% relative humidity). After silanization, the sample was removed from the solution and air-dried. The procedure was optimized using either a coating solution of only OTS or a binary mixture containing both MTS and OTS. The optimized experimental conditions included silanization time, silane concentrations/ratios, and humidity. Different levels of relative humidity were achieved using an Acrylic box where fine mists of water were sprayed onto the interior walls to increase the relative humidity. Conversely, dry nitrogen gas was used to decrease the humidity relative to the laboratory conditions. The sample was dried in an oven at 55 °C for at least 24 h prior to placing the substrate in the controlled environment. The sample was then placed in the artificial environment and allowed to incubate for 1 h before silanization. All samples were prepared in triplicates and characterized independently.

AgNP with a concentration of ~30 ppm was synthesized by adding 150 mL of 1.0 mM AgNO_3_ dropwise into an ice-cold solution containing 350 mL of 2.0 mM NaBH_4_ at a rate of 1 drop/s. The solution was stirred vigorously during the addition. When the addition was completed, stirring was stopped immediately. The resulting yellow solution was characterized using a QE65000 Scientific-grade Spectrometer (Ocean Optics Ltd., Winter Park, FL) to obtain the UV spectra and the SPR peak wavelength. Particle size analysis was performed using a Zetasizer Nano ZS system (Malvern Panalytical Ltd., Malvern, UK).

Various methods were tested for impregnating AgNP on cotton; the first method consisted of performing the silanization reaction and immediately transferring and immersing the substrate in another solution containing 10 mL of AgNP for 10 min. A second method consisted of first immersing the substrate in 10 mL of AgNP for 10 min and then air drying followed by immersion in the silane coating solution. A third method consisted of performing the silanization reaction followed by spraying 250 μL/cm^2^ of the AgNP colloid onto the surface before air drying and then further drying in an oven at 55 °C for 4 h before performing further experiments.

### Characterization and instrumentation

Water contact angle measurements were performed using an optical goniometer (VCA Optima video contact angle system, AST Products Ltd., Billerica, MA). Static contact angles were measured using 1.0 μL deionized water droplets. A total of 10 contact angles were measured per sample at different locations with each sample being prepared in triplicates (30 water contact angle values total).

Scanning electron microscopy was performed to image the cotton surfaces with a Nova NanoSEM 430 system (FEI company, Hillsboro, OR). EDS analysis was performed with the same instrument using an EDAX detector. All samples were sputtered with gold (Hummer 6.2 Sputtering System, Anatech USA, Hayward, CA) before imaging. Infrared spectroscopy measurements were performed using an ATR FT-IR spectrometer (UATR Spectrum Two, Perkin Elmer, Billerica, MA). The pure OTS silicate samples for control measurements were synthesized by reacting 200 μL OTS with 1.8 mL of deionized water. The MTS/OTS binary silicates were prepared similarly by adding a mixture of 100 μL MTS and 100 μL OTS to 1.8 mL of water. The products were subsequently collected using vacuum filtration and dried in an oven at 55 °C before characterization.

A modified form of the Kirby-Bauer disk susceptibility test was performed on AgNP impregnated cotton^24^. Luria-Bertani (LB) agar media was used as the culture medium for the test. Gram-negative *E. coli* K-12 was used as the test organism for bacterial susceptibility. The *E. coli* concentration was adjusted based on a 0.5 McFarland standard before inoculating. The modified cotton fabrics were cut into 6 mm diameter disks and placed onto the LB agar media after inoculating with *E. coli*. The plates were then incubated at 37 °C for 16 h before imaging. The susceptibility tests were performed in triplicates as well.

## Supplementary information

Supplementary information
