# Supplementary material for: Binary Silanization and Silver Nanoparticle Encapsulation to Create Superhydrophobic Cotton Fabrics with Antimicrobial Capability
Source: Sci Rep. 2019 Jun 24;9:9172. doi: 10.1038/s41598-019-45622-0 (PMC6591378; doi:10.1038/s41598-019-45622-0)
Supplement: Supplementary file 1 — Supplementary information [file 41598_2019_45622_MOESM1_ESM.pdf]

Supplementary material

for

Binary Silanization and Silver Nanoparticle Encapsulation to Create Superhydrophobic Cotton Fabrics with Antimicrobial Capability

William Shen,[a] Lishen Zhang,[a] Xiaochun Li,[*,b] and Hua-Zhong Yu[*,a,b]

[a] *Department of Chemistry, Simon Fraser University, Burnaby, British Columbia V5A 1S6, Canada*
[b] *College of Biomedical Engineering, Taiyuan University of Technology, Taiyuan, Shanxi 030024, China*

Additional experimental data for the optimization of relative humidity, silanization time, and OTS concentration on cotton fabrics; characterization of synthesized AgNP; ATR FT-IR of OTS treated cotton; EDS characterization of MTS/OTS treated cotton and SEM characterization of OTS/AgNP treated cotton fabrics.

----

* Corresponding author: hogan_yu@sfu.ca (H.Y.); lixiaochun@tyut.edu.cn (XL)

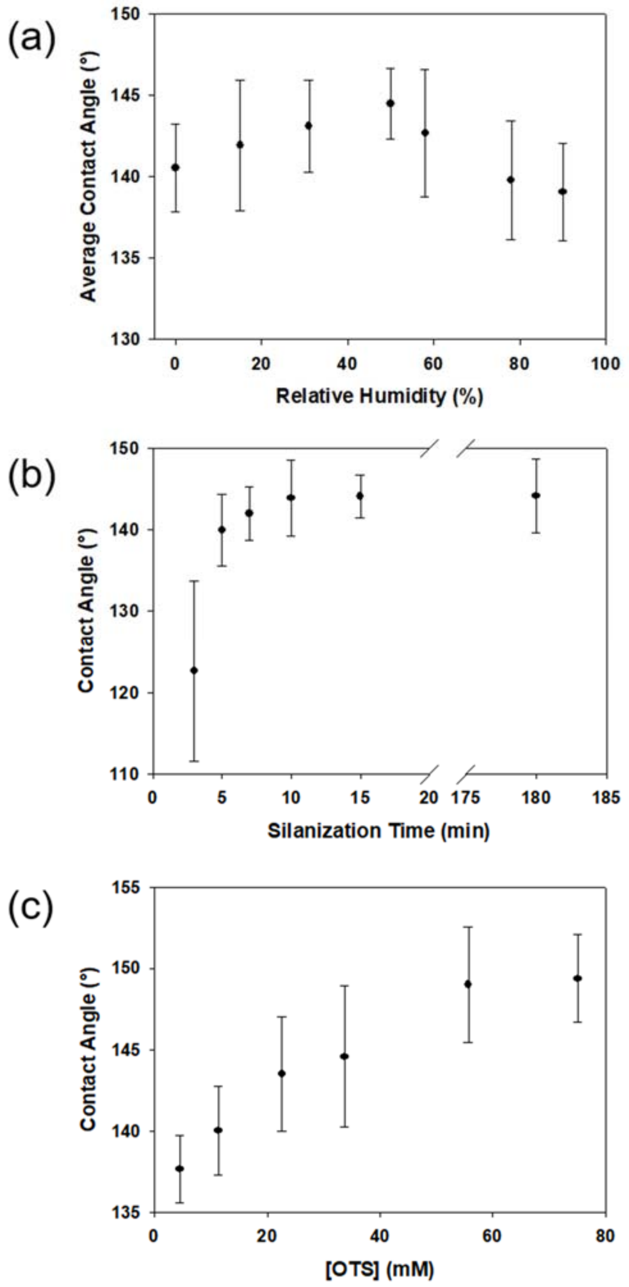

Fig. S1. Contact angle measurements of OTS-treated cotton fabrics with (a) different relative humidities, (b) silanization time, and (c) concentration of OTS. Cotton was treated using a solution-immersion process with OTS only in toluene. Contact angles were determined using a goniometer to measure 1.0 μL drops of Milli-Q water. The optimal relative humidity was 50% and optimal silanization time was 10 min. Treatments using concentrations greater than 20 mM OTS caused the cotton to become fragile where solid particles of silane were also visible to the naked eye.

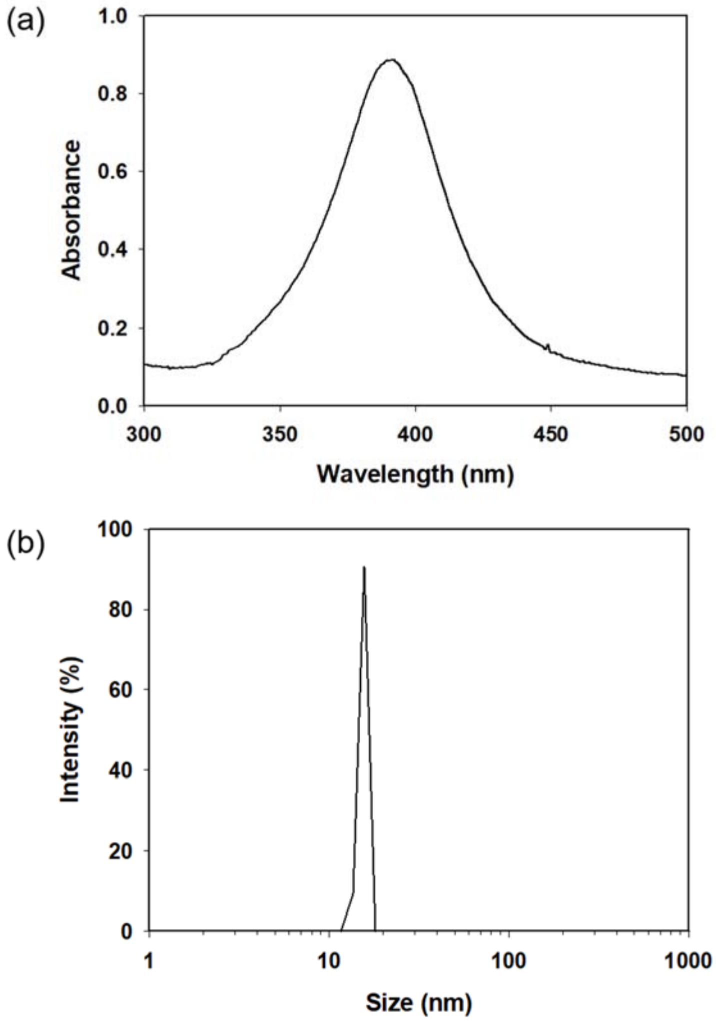

Fig. S2. (a) UV-Vis spectrum of the AgNP suspension. A surface plasmon resonance peak is shown at 391 nm. (b) Particle size analysis of the AgNP suspension using dynamic light scattering (DLS) measurements. The average particle size was 15 nm in diameter and had a tight particle size distribution.

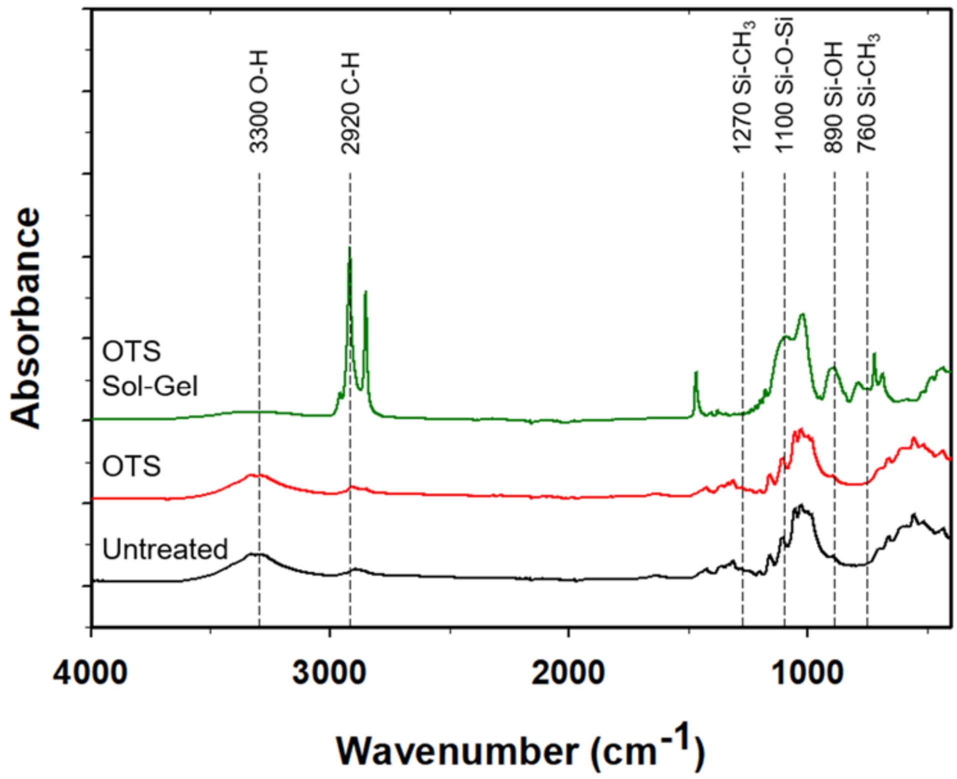

Fig. S3. ATR FT-IR of untreated cotton fabric (black trace), OTS (red trace), and a pure sol-gel derived from OTS (green trace). Both the OTS treated and untreated cotton samples had similar spectral features.

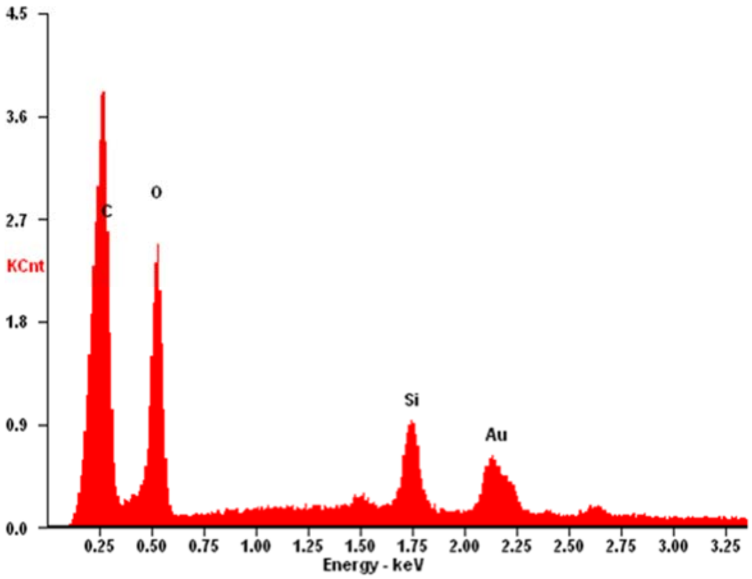

Fig. S4. EDS spectrum of MTS/OTS treated cotton fabrics. The Si K_α peak was observed at 1.74 keV indicating presence of Si-containing species (presumably polymethylsiloxane).

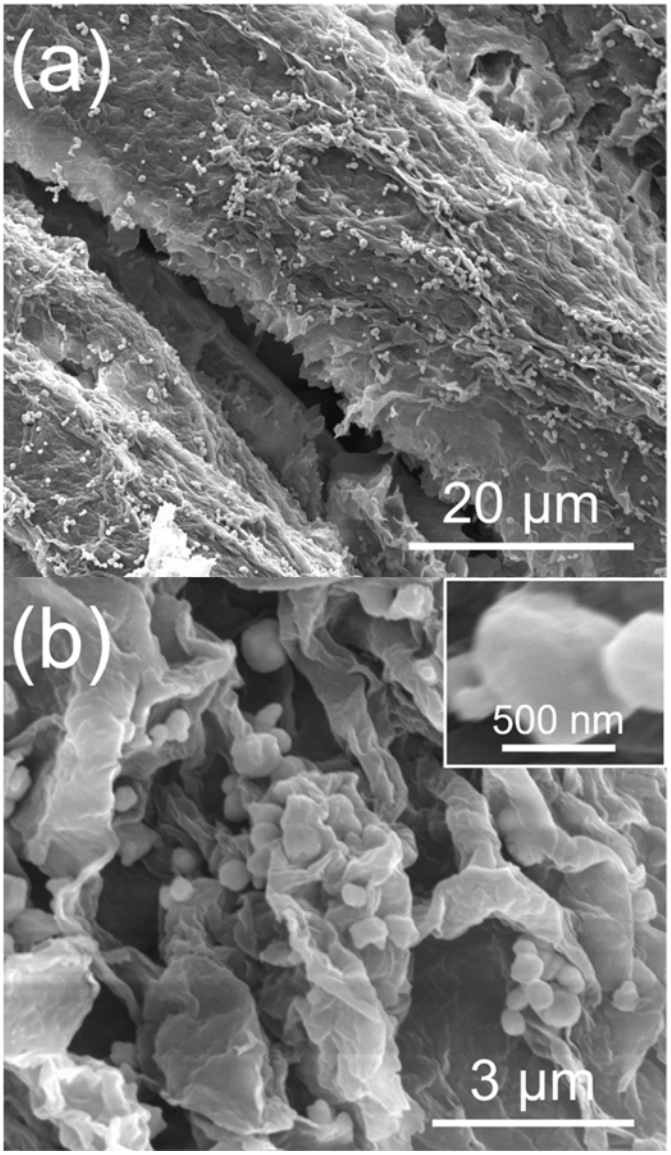

Fig. S5. SEM micrographs of OTS/AgNP treated cotton fabrics at different magnification. A large number of nanospheres are visible, which is different from rather smooth surface upon modification with a binary solution of OTS/MTS with a much lower total silane concentration. (a), (b), and the inset are presented to show different magnifications.
